# Livestock grazing supports native plants and songbirds in a California annual grassland

**DOI:** 10.1371/journal.pone.0176367

**Published:** 2017-06-14

**Authors:** Sasha Gennet, Erica Spotswood, Michele Hammond, James W. Bartolome

**Affiliations:** 1 The Nature Conservancy, San Francisco, California, United States of America; 2 San Francisco Estuary Institute, Richmond, California, United States of America; 3 East Bay Regional Park District, Oakland, CA, United States of America; 4 Department of Environmental Science, Policy, and Management, University of California, Berkeley, California, United States of America; Universidade de Lisboa Instituto Superior de Agronomia, PORTUGAL

## Abstract

Over eight years we measured the effects of plant community composition, vegetation structure, and livestock grazing on occurrence of three grassland bird species—Western Meadowlark (*Sturnella neglecta*), Horned Lark (*Eremophila alpestris*), and Grasshopper Sparrow (*Ammodramus savannarum*)—at sites in central California during breeding season. In California’s Mediterranean-type climatic region, coastal and inland grassland vegetation is dominated by exotic annual grasses with occasional patches of native bunchgrass and forbs. Livestock grazing, primarily with beef cattle, is the most widely used management tool. Compared with ungrazed plots, grazed plots had higher bare ground, native plant cover, and vertically heterogeneous vegetation. Grazed plots also had less plant litter and shorter vegetation. Higher native plant cover, which is predominantly composed of bunchgrasses in our study area, was associated with livestock grazing and north-facing aspects. Using an information theoretic approach, we found that all three bird species had positive associations with native plant abundance and neutral (Western Meadowlark, Grasshopper Sparrow) or positive (Horned Lark) association with livestock grazing. All species favored flatter areas. Horned Larks and Western Meadowlark occurred more often where there were patches of bare ground. Western Meadowlarks and Grasshopper Sparrows were most common on north-facing slopes, suggesting that these species may be at risk from projected climate change. These findings demonstrate that livestock grazing is compatible with or supports grassland bird conservation in Mediterranean-type grasslands, including areas with high levels of exotic annual grass invasion, in part because grazing supports the persistence of native plants and heterogeneity in vegetation structure. However, conservation of low-lying grasslands with high native species presence, and active management to increase the abundance of native plant species are also likely to be important for sustaining grassland birds long-term.

## Introduction

Grasslands are among the most highly converted and least protected of all terrestrial habitats, and grassland songbird communities throughout North America and the western United States are declining faster than any other comparable bird guild [1–4]. While the causes of these declines remain poorly understood, conversion of suitable habitat has been cited by many as a factor, and California’s grasslands are being converted faster than rates of protection [3, 5]. A similar pattern of land use intensification and grassland species decline is occurring in other Mediterranean regions, as well, including southern Europe [6, 7].

Not all grasslands are alike in their ability to support these species, however. Terrestrial birds select habitat to forage and breed based on factors at multiple spatial scales [7, 8]. Therefore, effective conservation prioritization and management must be informed by landscape and finer-scale habitat associations, as well as an understanding of how grassland management practices, such as livestock grazing, affect habitat.

At a landscape scale, patch size, land cover type richness and composition, and proximity to development have been shown to be influential factors for this avian guild in California’s Mediterranean grasslands [9]. Fine-scale structural characteristics of vegetation, including height, presence of bare ground and dead aboveground plant material (“litter”) can also influence site occupancy and abundance, but these characteristics have not previously been well studied in this type of grassland [10, 11]. Structural characteristics can influence foraging efficiency [12, 13], nest site availability [14, 15], and predator protection [16–18]. Local vegetation is strongly influenced by topographic variables in grasslands as well as climatic factors [19–22]. In Mediterranean- type, semi-arid grasslands in California where water is a primary limiting resource, site-specific topography can also affect both productivity and community composition, but again their influence on bird habitat quality has not previously been studied [23].

As in other Mediterranean and semi-arid regions globally, livestock grazing is a widespread land use in the western United States on both private and public lands. Grazing objectives often include, in addition to food production, managing invasive plant species [24] and controlling fuel buildup [25]. Although California grasslands have been grazed for dairy, wool, and meat production for centuries, and for fuels and vegetation management for decades, the effects of livestock grazing on grassland birds have only rarely been investigated in California. Grazing by livestock can alter both the structure [26–28] of vegetation and the species composition [29–33]. Typical grazing practices in California’s extensive rangelands, including in this study area, include low to moderate stocking densities, long rotations among pastures and/or seasonal use, and minimal supplemental feeding. As a result, herbivory varies in space and time, increasing the spatial and temporal heterogeneity of vegetation structural properties and creating a mosaic of disturbance across the landscape [26, 34–37]. Selective foraging, removal of grass and dead plant material and trampling can all alter the fine-scale structure of vegetation in ways that can either be beneficial or detrimental to grassland birds, depending on the particular breeding and foraging requirements of each species [10].

Regions around the world with Mediterranean climates, including California, are recognized as global biodiversity hotspots [38–40]. Changes in land use and management have impacted, and continue to threaten, ecological communities, native species, and environmental quality. In California, European settlement, including the introduction of livestock grazing, led to one of the most dramatic biological invasions on earth, in which millions of hectares of grasslands shifted from diverse native forbs and perennial grasses to dominance by a smaller number of exotic annual grasses, many of southern European (Mediterranean basin) origin [41–44]. This alteration resulted in cascading changes to fine-scale habitat characteristics, natural community composition, and ecosystem function, including nutrient and water cycling, invertebrate and pollinator composition and abundance, and homogenized vertical vegetation structure [45–51]. Understanding how these changes affect habitat suitability is critical to conserving grassland bird species in California [52, 53].

In the Diablo Range of central California, where our study was located, the grassland songbird guild is dominated by three species: Western Meadowlark (*Sturnella neglecta*), Horned Lark (*Eremophila alpestris*), and Grasshopper Sparrow (*Ammodramus savannarum*). The distributions and local habitat requirements of these species are poorly understood across most of the state [9, 54–56]. Studies in other grassland regions of the United States suggest that each species has somewhat different fine-scale habitat requirements, in keeping with their unique life history traits, and that species-specific habitat requirements can vary between populations and regions due to different climatic patterns and vegetation (e.g., [57–60]. For example, California’s Mediterranean grasslands are climatically and structurally different from prairies in the Midwestern United States, where many previous studies have been conducted [61, 62]. In fact, they are more similar in terms of plant species composition, climate, and vegetation structure to other Mediterranean regions, including southern Europe, than to other North American grasslands. Therefore, regional empirical studies that explore habitat utilization for these species are critical to accurately understand their requirements and to determine appropriate land use and management.

In this study, we examined the relationship of fine-scale habitat characteristics, including vegetation structure and native plant abundance, and livestock grazing- the dominant land use in remaining California rangelands today- with the occurrence of these three ground-nesting grassland songbirds at four sites over eight years. Our main objectives were to 1) determine whether the structural characteristics and plant species composition of grassland vegetation varied between grazed and ungrazed plots and 2) evaluate whether vegetation, structural characteristics, and livestock grazing influence utilization by grassland birds.

## Methods

### Study area

A total of 36 plots were sampled at four sites in the northern Diablo Range, or inner Coast Range, east of the San Francisco Bay (Table 1). All sites, which included Morgan Territory (1900 ha), Pleasanton Ridge (2,130 ha), Sunol-Ohlone (6,715 ha) and Vasco Caves (665 ha), are publicly-owned open space lands in Alameda or Contra Costa counties and are managed by the East Bay Regional Park District (EBRPD) for conservation, biodiversity and public recreation. Weather patterns in the project area are characteristic of Mediterranean regions, with cool, wet winters, hot, dry summers, and high inter-annual variability in timing and amount of precipitation.

**Table 1:** Number of plots sampled during study period from 2004–2011. All plots were sampled for birds three times each year and for vegetation structure and composition. Number of plots are listed by site and year with number of “Grazed” plots first and (“Ungrazed”) plots following in parentheses. Sunol and Ohlone sites are contiguous.

Year	Morgan Territory	Pleasanton Ridge	Sunol-Ohlone	Vasco Caves	Total plots
2004	3 (3)	6	0	10	19 (3)
2005	5 (5)	6	6 (3)	6 (4)	23 (12)
2006	5 (5)	6	6 (3)	6 (4)	23 (12)
2007	5 (5)	6	6 (3)	6 (4)	23 (12)
2008	5 (5)	6	6 (3)	6 (5)	23 (13)
2009	5 (5)	6	6 (3)	6 (5)	23 (13)
2010	5 (5)	6	6 (3)	6 (5)	23 (13)
2011	5 (5)	6	6 (3)	6 (5)	23 (13)

UC Berkeley’s Rangeland Ecology Laboratory, led by co-author Bartolome, and where all other authors were associated at the time of the sampling, had permission and funding from EBRPD to sample at these sites.

### Plot selection

The sample plots were located at least 200 m apart within a two-level stratified random design based on: 1) distance of at least 200 m from non-grassland land-cover type to avoid influence from other vegetation types and anthropogenic structures, and 2) livestock grazing (ungrazed, grazed). Vegetation and bird sampling used the same plot centers and were followed over 8 consecutive years.

### Livestock grazing and fire

A total of 23 grazed plots were sampled. These were areas stocked by livestock (beef cattle or sheep) at low to moderate rates typical of this region, averaging 3 Animal Unit Months (AUM) /ha. One AUM is equivalent to one cow and calf grazing for one month. [63]. Management of the grazed grasslands consisted of ensuring more than 1,000 lbs./acre (1,121 kg/ha) of residual dry matter (RDM) was left in each field in September, after the end of the growing season and prior to the start of winter rains. The timing, intensity of grazing, kind and class of animal (e.g., beef cattle compared to sheep), and utilization are important considerations in grazing management; RDM serves as an effective index for these factors [64]. Ungrazed plots within Morgan Territory and Sunol-Ohlone were located in areas where livestock grazing was removed at least 20–30 years prior to the study. Ungrazed plots at Vasco Caves, on the other hand, were fenced for resource management by EBRPD in 2004 after our first year sampling was completed. Since those plots were ungrazed for the majority of the study period, we classified them as ungrazed. All of the land in this study, like most of the grassland and oak savanna in the Mediterranean region of California, had likely been stocked by domestic grazing animals for extended periods during the last century or more. Detailed historical grazing records are not available. There are more grazed than ungrazed plots in the study because of this very widespread use of grazing as a land management tool in California’s grasslands—too few large grassland areas remain ungrazed for long periods for a balanced sampling design in the study area. In addition, several plots that had been ungrazed at the initiation of the study were grazed later due to management decisions by the landowner.

None of the sites where our surveys occurred use prescribed burning for grassland management. However, Vasco Caves was the location of a wildfire in fall 2006. Three plots were affected; no lasting vegetation change was detected.

### Vegetation and plot variables

We recorded vegetation data with four 17 m line-point transects arranged in cardinal directions (N, E, S,W) around the permanently-marked plot center [65]. We recorded the vegetation species and height of the first-foliar-hit at each point, located by lowering a sharpened point into the canopy. Plant “hits” were taken every 10 cm for 4.5 m; beyond 4.5 m, hits were recorded every 50 cm for a total of 70 hits per transect and 280 points/ plot/ year. If no plant was encountered when lowering the point for a hit, the material encountered on the ground (e.g., rock, bare ground) was noted and the hit was recorded as height of zero. “Litter,” defined as the previous year’s biomass, was also recorded if encountered. All biomass not considered litter, i.e. this year’s growth either in the thatch or standing dead layer, was identified to plant species. The line-point transect method may over-sample the tallest vegetation and the dominant species relative to quadrat-based methods that rely on ocular estimates [66, 67], but has the advantage of reducing among-observer variation. Thus, while species richness and abundance of rare species are likely to be conservative, estimates of abundance are more robust to observer bias, which is an advantage for long term-studies where personnel may change from year to year [66].

Variables for native plant cover, litter, and bare ground were calculated as the percent absolute cover of each plot (total hits as percent of 280 points). Vegetation height was the plot average height of all points. Vertical heterogeneity of vegetation was calculated as the plot-level coefficient of variation of vegetation height of all points.

We included topographic variables slope and a measure of aspect (compass direction, which relates to incoming solar radiation) in the models of bird occupancy. In California grasslands, solar radiation is lower on north-facing slopes, which leads to lower air and soil temperatures [68] and higher soil moisture [69] on north-facing exposures. Aspect was converted into northness using the formula:

$$Northness=Cosine(\frac{Aspect*\pi }{180})$$

This calculation yields values that vary from -1 (south) to 1 (north) and quantify the degree to which the aspect value is north [70], and is commonly used in ecology [71–74] to approximate the amount of solar radiation reaching a site due to the strong link between aspect and amount of incident radiation. All grassland bird, vegetation, and topographic data are found in Table A in S1 File.

### Birds

Ten-minute point count surveys were conducted at each plot three times during the breeding season (15 March- 10 June), with a minimum of ten days between sampling dates [75, 76]. Our analysis only included visual or aural observations recorded within 100 m of the plot center to minimize inaccuracy associated with identification at greater distances [77]. Occurrences were coded as present if at least one individual of a species was observed at a study site in at least one of the three visits for a corresponding year, and as zero otherwise. This approach, as well as a 100 m distance cutoff for detection, is conservative for estimating occupancy. We chose a conservative method because an occupancy modeling analysis approach was not possible due to the nested and temporally replicated design of our study, which required the inclusion of random effects to account for the lack of independence of data collected within the same year and at the same site [78–80].

### Statistical analysis

To evaluate whether grazing and native cover influenced vegetation structure, we used linear mixed models (LME) with site nested within year as random effects. This error structure was used in order to account for the nested and temporally repeated design of the study [81]. We chose to include site and year as fixed effects to account for the lack of independence of data collected within the same site and during the same year. At the same time, we were primarily interested in the effects of vegetation and grazing on bird abundance, and therefore chose not to include either site or year as fixed effects. We fit separate models for each of the dependent variables vegetation height, vertical heterogeneity, litter, and bare ground. Fixed effects included grazing, native cover, northness, and slope. We fit 16 separate models including a saturated model, an intercept-only null model, and all possible combinations of fixed effects terms (including each fixed effect alone). We chose to fit all possible combinations of models because while Burnham and Anderson (2002) advocate selecting biologically relevant models *a priori*, we had little reason to suspect that any particular combinations of fixed effects were more likely than any other. Testing all possible models is not the ideal strategy in model selection. However, it is a practical approach when there is insufficient *a priori* information to develop a reduced set of plausible candidate models (e.g., [82]. For example, in our study, the height of vegetation is equally likely to be related to grazing, northness and native cover as it is to any other combination of parameters, including grazing, slope and northness. We square root transformed the variables bare ground and litter and log transformed vertical heterogeneity before analysis in order to meet model assumptions.

We used generalized linear mixed modeling (GLMM) to analyze the relationship between the occurrence of birds and site, vegetation, and grazing variables with a binomial error structure. We identified a candidate model set that included 20 models *a priori* following guidelines outlined in [83]. Models included fixed factors with vegetation structural variables (vegetation height (cm), litter and bare ground), one vegetation composition variable (native plant cover), topographic site variables (northness and slope), and whether the site was grazed or ungrazed during the study period. The candidate set of models included a single fully saturated model, a null intercept-only model, and every variable on its own. In addition, 11 reduced models contained subsets of variables including a structure-only model, a topography-only model, a grazing and structure model, and a grazing and topography model. We used variance inflation factors and Spearman rank correlations before analysis in order to assess whether variables were collinear with each other. We considered a variance inflation factor over 10 and Spearman rank correlations over 0.5 to be problematic [78]. We eliminated vertical heterogeneity from all models on the basis of its correlation with height (*r* = 0.663). We retained the variable in the analysis of the effects of grazing on vegetation because of its known importance to grassland birds, and we can infer some of its effects on birds through its association with grazing despite its removal from models of bird occurrence.

For both vegetation structure and bird occurrence we compared models using Akaike’s Information Criteria corrected for small sample sizes (AIC_c_), using AIC_c_ weights (*w*_*i*_). We determined a top performing candidate set of models for each variable within AIC_c_< 4, and we used model averaging to obtain parameter estimates. In the analysis of bird occupancy, we used AICc to calculate importance weights for all variables included in the final candidate set of models for each species. Because variables were not included in equal numbers in the original model set, we standardized importance weights by dividing the weight values by the number of times each variable was included in the original model set, and then multiplying by the average number of times variables were included in models. This calculation relativizes the importance weight values, making them comparable across variables and species. For bird occurrence models, we estimated parameters using Restricted Maximum Likelihood (REML) estimation and the glmer function in the lme4 package in R 2.12.1 [84] following recommendations in Bolker et al. [79] and Zuur et al. [80]. For linear mixed models of vegetation structure, we used the nlme package [85] with Maximum Likelihood estimation.

To evaluate the variance explained by models we calculated R^2^ values for all models in the 95% confidence set of models for each bird species using the methods of Nakagawa and Schielzeth [86]. We calculated the marginal R^2^ (R^2^_GLMM(*m*)_), which quantifies the variance explained by fixed factors and the conditional R^2^ (R^2^_GLMM(*c*)_), which quantifies variance explained by both the fixed and random factors.

## Results

**Fig 1:**
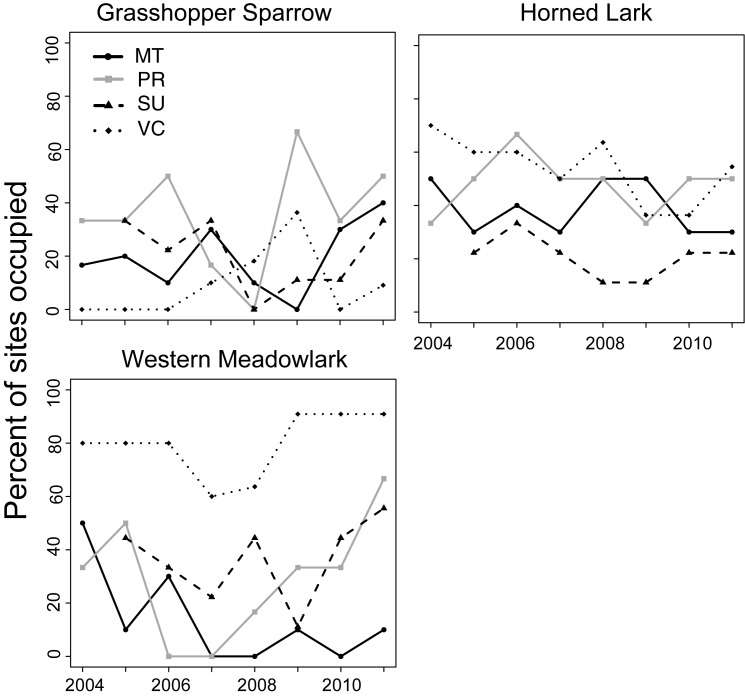
Occurrence of three grassland bird species during the study period from 2004 to 2011 in 36 plots in four sites in Mediterranean grasslands in California. Percent of plots occupied represent the percent of all plots where each species was found at least once in three point counts for a given site in a given year. Each line represents an individual site (park) including Morgan Territory (MT, black solid line, circle points), Pleasanton Ridge (PR, grey solid line, square points), Sunol-Ohlone (SU, black dashed line, triangle points), and Vasco Caves (VC, black dotted line, diamond points).

Two of the three focal species, Western Meadowlarks and Horned Larks, were observed frequently across the study area and consistently every year throughout the study period (Fig 1). Western Meadowlarks were the most frequently observed species, with an average of 3.6 ± 3.2 SD plots per site occupied in each year. Horned Larks occupied an average of 3.5 ± 1.6 SD plots per site per year, and Grasshopper Sparrows an average of 1.7 ± 1.3 SD. Plot occupancy varied widely, with some plots never occupied during any years, some occupied consistently every year, and variable occupancy in others. Total occupancy across all plots in a site (park) varied both among sites and across years (Fig 1).

### Grazing and vegetation

**Fig 2:**
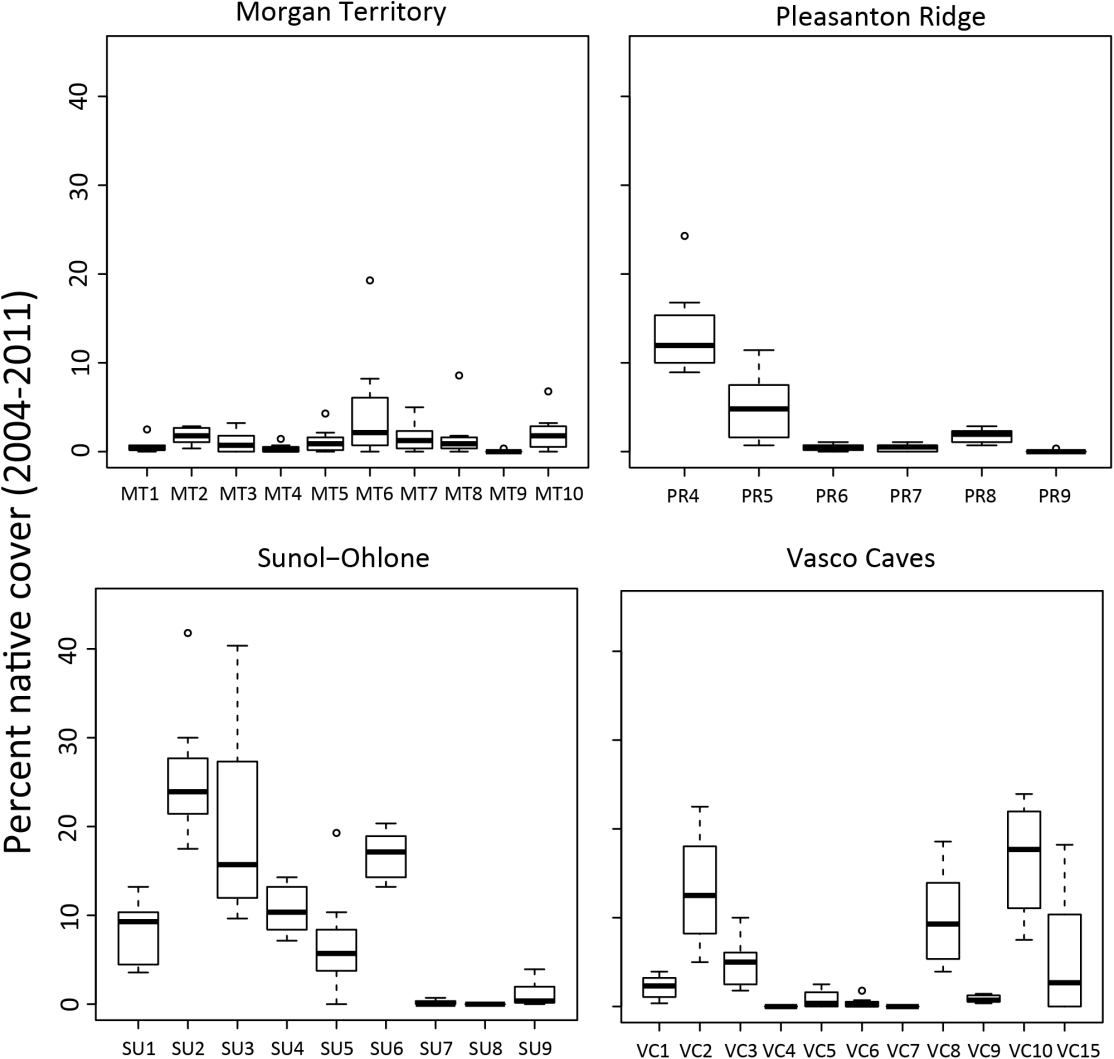
Native plant species cover during the study period from 2004 to 2011 in 36 plots in four sites in Mediterranean grasslands in California.

Native plant cover was variable across plots and sites, but relatively constant among years (Fig 2). Our study plots had a low range of native cover (0–20%) with the bunchgrass purple needlegrass (*Stipa pulchra*, alternate name *Nassella pulchra*) by far the most abundant native plant species. Other common native plant species were herbaceous forbs (wildflowers such as clovers [*Trifolium* spp.], Johnny jump ups [*Viola pedunculata*], dwarf checkerbloom [*Sidalcea malviflora*]). The four top dominant exotic species, Italian rye grass (*Festuca perrenis*, alternate name *Lolium multiflorum*), slender wild oat (*Avena barbata*), common wild oat (*Avena fatua*), ripgut brome (*Bromus diandrus*), were all annual grasses; foliar cover of these individual species ranged from 60–80% of each plot.

**Fig 3:**
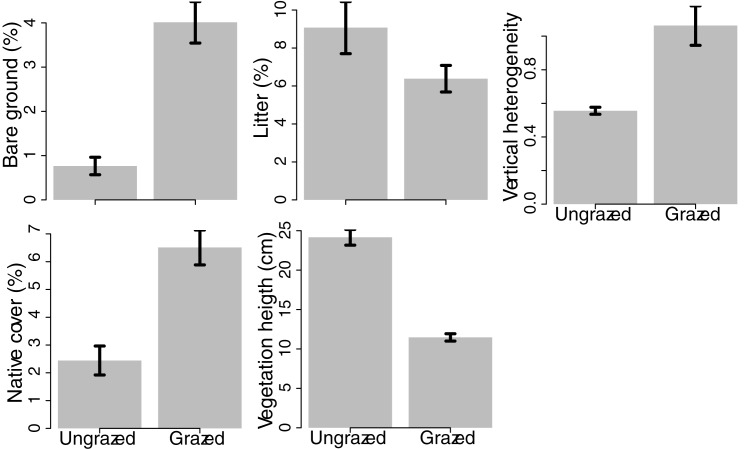
The effect of livestock grazing on vegetation structural properties measured along four line-point transects (70 points each) in four sites in Mediterranean grasslands in California. Vegetation structural variables include average vegetation height (cm), vertical heterogeneity (coefficient of variation of vegetation height), amount of bare ground (% of all hits), amount of litter (% of all hits), and native cover (% of all native vegetation hits). Bars represent averages (± 1SE) across all plots in the study from all years and across all four sites.

Livestock grazing was related to all structural vegetation variables (Tables 2 and 3, Fig 3). Grazing reduced vegetation height and litter accumulation, and increased bare ground, native cover, and vertical heterogeneity. Northness and slope also influenced vegetation structure. North-facing slopes were associated with less litter, shorter vegetation height, less bare ground, less vertical heterogeneity, and more native cover. Steeper slopes were weakly positively associated with all vegetation structure variables and native cover.

**Table 2:** Relationships between grazing, native cover, aspect (northness), slope and vegetation structural characteristics. Estimates are model averaged from top performing linear mixed models within ΔAIC_c_ < 4 from 16 possible models for structural variables and 10 possible models for native cover. Estimates include model averaged standard error and confidence intervals.

* *	Variable	Estimate	SE	Low CI	Upper CI
*Height (cm)*					
	Intercept	4.78	0.17	4.44	5.11
	Grazing	-1.36	0.09	-1.55	-1.18
	Native cover (%)	-0.02	0.01	-0.04	-0.01
	Northness	-0.23	0.07	-0.37	-0.09
	Slope	0.00055	0.0034	-0.01	0.01
*Vertical heterogeneity*					
	Intercept	0.70	0.06	0.58	0.82
	Grazing	0.20	0.045	0.11	0.29
	Native cover (%)	0.0046	0.0030	-0.0013	0.010
	Northness	-0.05	0.03	-0.11	0.019
	Slope	0.0018	0.0015	-0.0011	0.0048
*Litter (%)*					
	Intercept	1.96	0.58	0.81	3.11
	Grazing	-0.48	0.13	-0.74	-0.23
	Slope	0.012	0.0044	0.0037	0.021
	Northness	-0.10	0.09	-0.29	0.08
	Native cover (%)	0.0087	0.0087	-0.0084	0.026
*Bare ground (%)*					
	Intercept	-0.08	0.31	-0.69	0.53
	Grazing	0.76	0.12	0.51	1.00
	Native cover (%)	0.02	0.01	0.00	0.04
	Northness	-0.23	0.09	-0.40	-0.06
	Slope	0.018	0.0040	0.010	0.03
*Native cover (%)*					
	Intercept	1.15	0.23	0.68	1.61
	Grazing	0.78	0.18	0.42	1.14
	Northness	0.80	0.13	0.56	1.05
* *	Slope	0.0081	0.0063	-0.0044	0.021

**Table 3:** Model selection results for analysis evaluating the effects of vegetation structure, topography and grazing on the presence of three grassland bird species. Models are top performing model set of 20 candidate models fit using a generalized linear mixed model with site and year as random effects. Top candidate model set includes models within AIC<4 of the top performing model. Table includes the number of parameters (*k)* in each model, the change in AICc from the best performing model to all others,the AICc weight (*w)* or strength of evidence for each model, the marginal R^2^ (*R*^*2*^_GLMM(*m*)_), and the conditional R^2^ (*R*^*2*^_GLMM(*c*)_) values indicating the variance explained by the fixed effects (marginal R^2^) and fixed and random effects (conditional R^2^) terms in each model.

	Model covariates	K	LL	AICc	AICc change	wi	*R*^*2*^_GLMM(*m*)_	*R*^*2*^_GLMM(*c*)_
*Horned Lark*								
	Grazing + Aspect + Slope + Native cover + Litter + Bare ground + Vegetation height	10	-133.96	288.77	0.00	0.52	0.430	0.610
	Grazing + Native cover + Vegetation height + Bare ground	7	-137.66	289.75	0.98	0.32	0.420	0.560
	Grazing + Native cover + Vegetation height + Bare ground + Litter	8	-137.61	291.78	3.01	0.12	0.420	0.560
*Grasshopper Sparrow*								
	Aspect	4	-128.35	264.84	0.00	0.24	0.060	0.200
	Aspect + Native cover	5	-127.39	265.01	0.17	0.22	0.060	0.200
	Native cover	4	-128.98	266.10	1.26	0.13	0.040	0.140
	Aspect + Slope	5	-128.29	266.81	1.97	0.09	0.630	0.210
	Aspect + Native cover + Slope	6	-127.32	266.97	2.13	0.08	0.061	0.200
	Grazing + Native cover	5	-128.56	267.35	2.51	0.07	0.048	0.130
*Western Meadowlark*								
	Aspect + Slope + Native cover	6	-133.57	279.47	0.00	0.50	0.150	0.430
	Grazing + Aspect + Slope + Native cover + Litter + Bare ground + Vegetation height	10	-130.25	281.35	1.88	0.20	0.180	0.470
	Native cover	4	-137.28	282.72	3.25	0.10	0.090	0.410

### Birds

**Fig 4:**
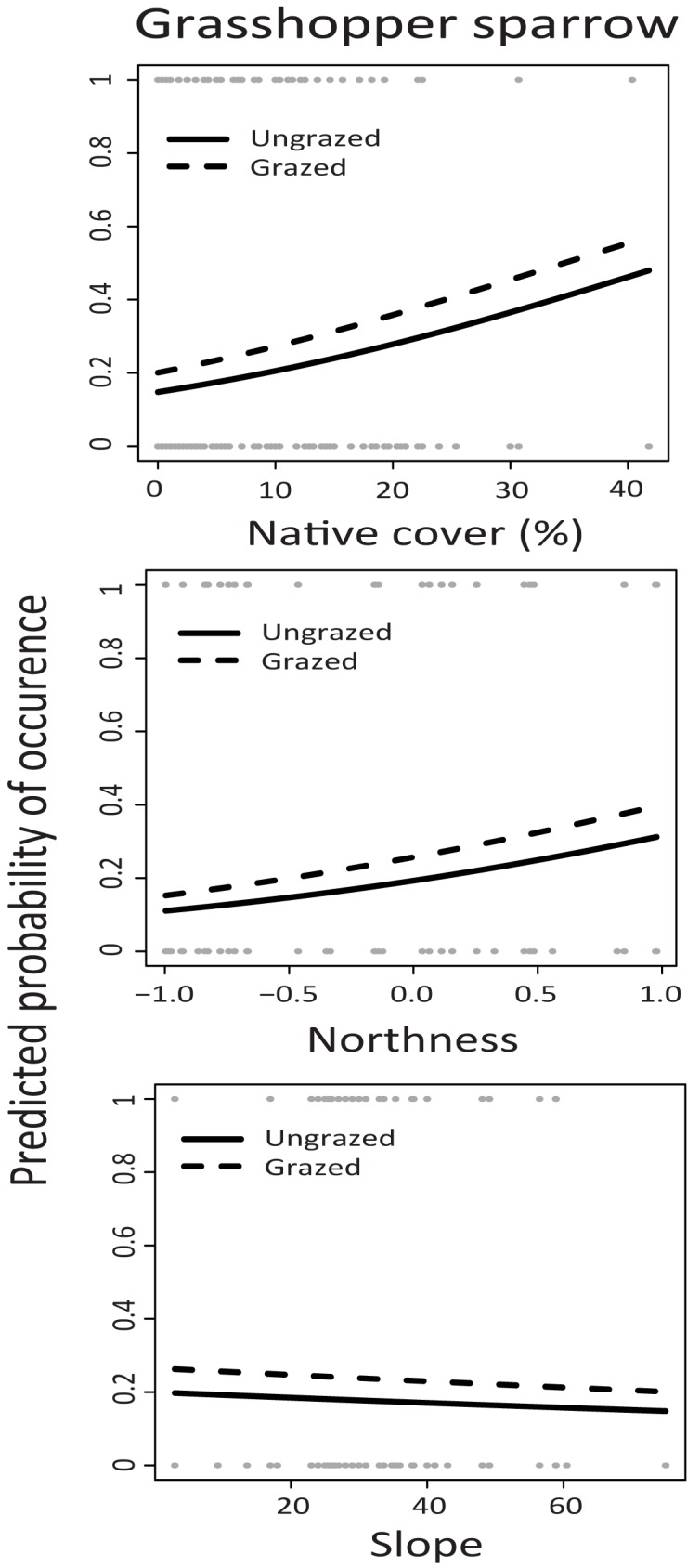
Grasshopper Sparrow predicted probability of occurrence as a function of Northness, native cover (%) and slope in grazed (dashed line) and ungrazed (solid line) plots. Results are taken from a single best fitting model (Table 3) for the occurrence from point count data collected at four sitess in Mediterranean grasslands in California sampled during the study period from 2004–2011. Raw data are shown in circles at top and bottom of figures.

**Fig 5:**
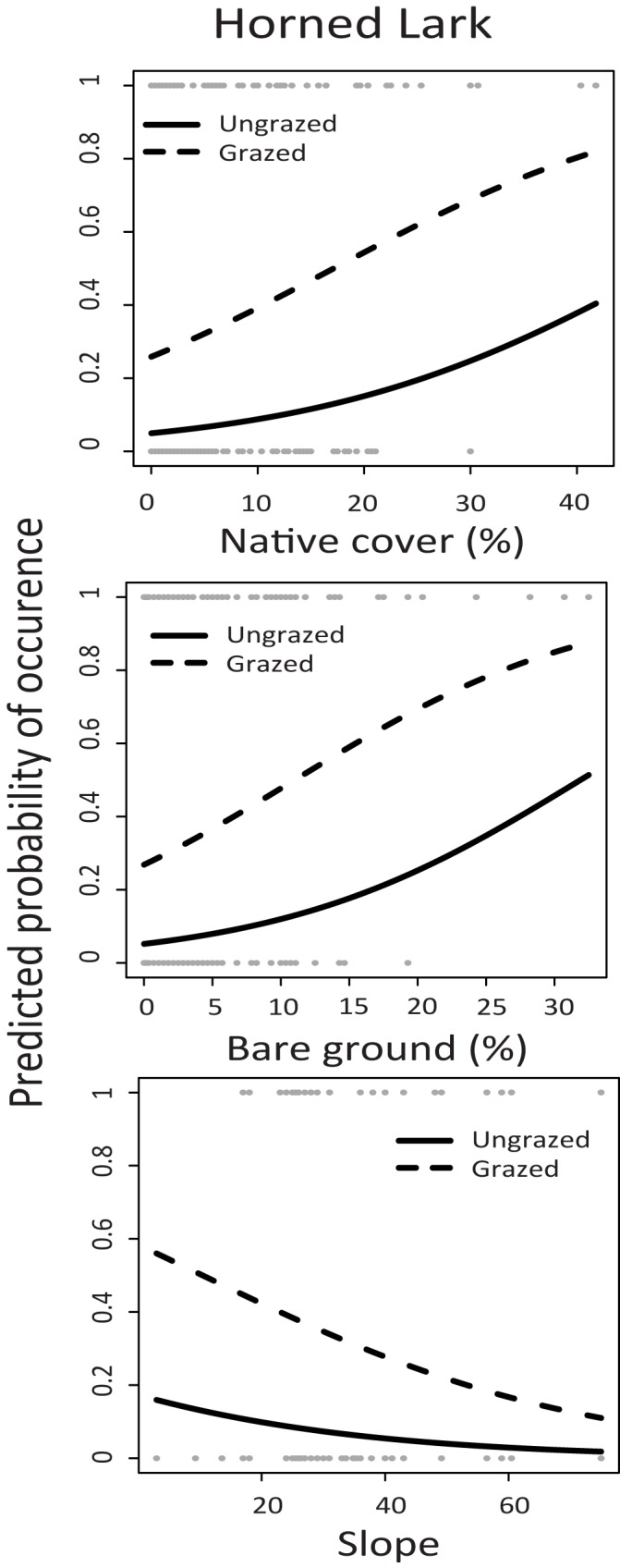
Horned Lark predicted probability of occurrence as a function of native cover (%), bare ground (%) and slope in grazed (dashed line) and ungrazed (solid line) plots. Results are taken from a single best fitting model (Table 3) for the occurrence from point count data collected at four sites in Mediterranean grasslands in California sampled during the study period from 2004–2011. Raw data are shown in circles at top and bottom of figures. Dashed lines represent plots with livestock grazing during the study period, and solid lines represent plots where there was no current livestock grazing.

**Fig 6:**
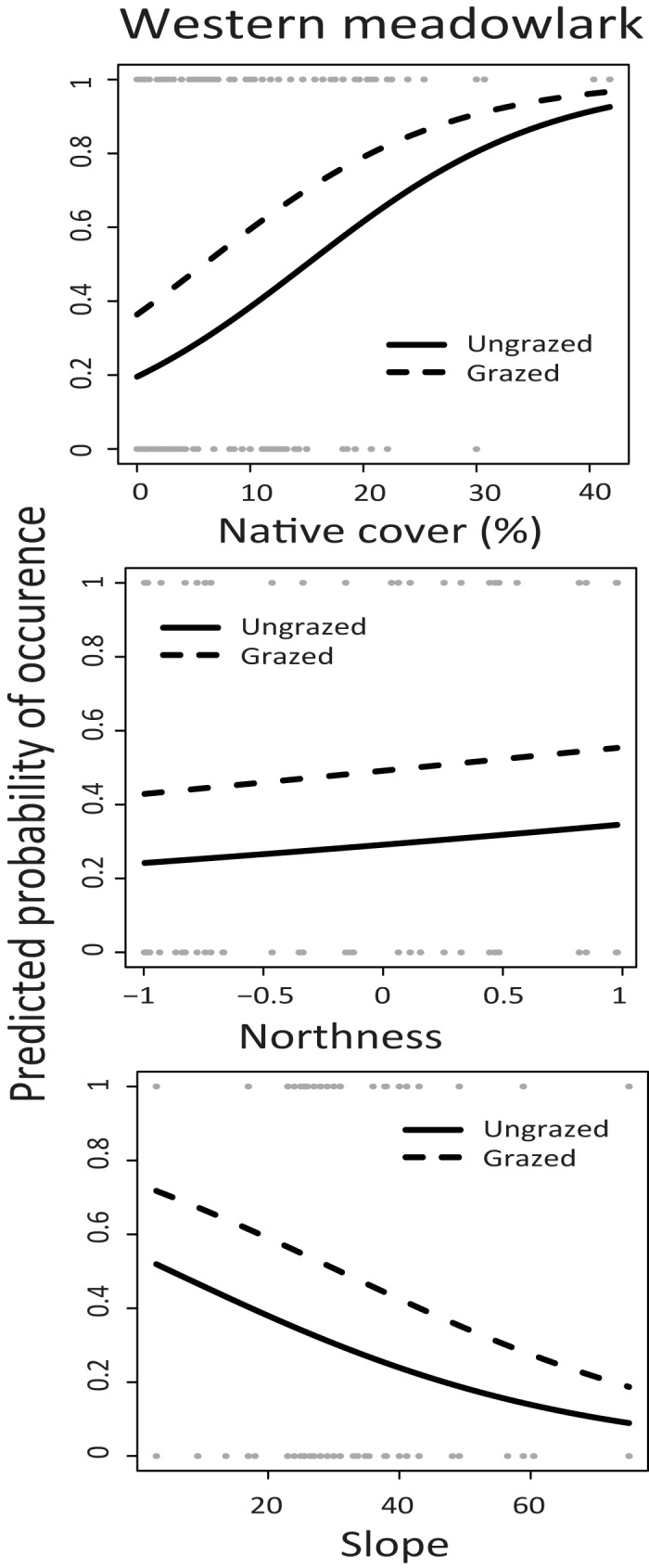
Western Meadowlark predicted probability of occurrence as a function of topographic slope (%), Northness and native cover (%) in grazed (dashed line) and ungrazed (solid line) plots. Results are taken from a single best fitting model (Table 3) for the occurrence from point count data collected at four sites in Mediterranean grasslands in California sampled during the study period from 2004–2011. Raw data are shown in circles at top and bottom of figures.

All three grassland bird species were found most often on plots with higher levels of native plant cover and low to flat terrain (Tables 3 and 4, Figs 4–6). Grasshopper Sparrows were the least frequently observed focal species in the study, and were found most often on plots with higher native cover, a more northerly aspect, and lower slopes (Tables 3 and 4, Fig 4). In addition, Horned Larks were found more often on plots with livestock grazing, and more bare ground, (Tables 3 and 4, Fig 5). There was a weak increase in Western Meadowlark presence with more bare ground (Tables 3 and 4, Fig 6). Only Horned Larks were strongly associated with grazing, although grazing was included in the confidence set of models for all species.

**Table 4:** Importance weights (*w+)* for variables included in models for three species of grassland birds. *N* is the number of models and *w+* is the summed Akaike weights for all models sharing a given model structure from a candidate set of 20 models for each species. Importance values (*w+*) represent values standardized by the number of models in which each variable occurs. The top three most important variables for each species are highlighted in bold.

Covariate	*N*	Grasshopper Sparrow	Horned Lark	Western Meadowlark
Bare ground	9	0.073	**0.791**	0.236
Grazing	8	0.155	**0.892**	0.274
Vegetation height	10	0.075	0.713	0.213
Litter	6	0.074	0.773	0.312
Native cover	8	**0.514**	**0.853**	**0.890**
Northness	5	**0.934**	0.742	**1.060**
Slope	4	**0.353**	**0.927**	**1.243**

Models generally captured the most variation in the data for Horned Larks, followed by Western Meadow Larks. Models for Grasshopper Sparrows performed the least well of the three species; the top performing model explained only around 20% of the variation in the data (R^2^_GLMM(*m*)_ = 0.2, Table 3), of which aspect explained only 6% (R^2^_GLMM(*c*)_ = 0.61, Table 3). Around 61% of the variation in the data was explained by the top performing model in Horned Larks (R^2^_GLMM(*m*)_ = 0.61, Table 3), of which 43% was jointly explained by fixed factors grazing, aspect, native cover slope, litter, bare ground and vegetation height (R^2^_GLMM(*c*)_ = 0.43, Table 3). The top performing model for Western Meadowlark occupancy explained 43% of the variation in the data (R^2^_GLMM(*m*)_ = 0.43, Table 3), with fixed factors aspect, slope and native cover jointly explaining 15% (R^2^_GLMM(*c*)_ = 0.18, Table 3). The random factors park and year generally explained between 15 and 25% of the variation in data for each of the three species (Table 3).

## Discussion

Our overarching finding is that breeding season occurrence of these three grassland bird species in Mediterranean-type grasslands in California is influenced by fine-scale vegetation structure, plant species composition, and topographic factors, and that livestock grazing is a compatible or beneficial use. Vegetation structure and composition, including abundance of native plants and presence of bare ground patches, are influenced by livestock grazing and could potentially be further enhanced through grazing management and/or active restoration. Topographic factors like slope cannot be influenced by management activities or livestock operations, but can be factored into conservation planning, land use policy, and land protection strategies. Our findings are in keeping with findings for grassland species across the western United States [87] and may hold useful information for avian conservation in the Mediterranean region of southern Europe, as well, where grassland bird habitat preferences have not been extensively studied over time [7].

Across the study area, occupancy levels were relatively low, especially in areas where native species cover was low. This suggests that Mediterranean grasslands in California with the highest levels of exotic invasive cover provide adequate but not high quality habitat for grassland birds, especially absent livestock grazing, and supports the conclusion that there is a need for active restoration of native plant species for grasslands birds to persist. Furthermore, for all three species, substantial variation in occupancy was related to the park and year in which surveys were performed, highlighting the strong (but not atypical) role of spatial and temporal variability in determining occupancy. Finally, while aspect, native cover and slope were related to the occupancy of Grasshopper Sparrows, these factors explained a relatively small amount of the variation in occupancy suggesting that additional factors that were not included in this study could be equally or more important for this species.

Other published studies of the effect of nonnative plants on reproduction and abundance of grassland birds have yielded mixed results with some finding positive associations [45, 88–90], while others have found lower abundance and occupancy of grassland sites dominated by introduced plant species [2, 89]. Our study supports studies of Western Meadowlarks in other grassland types which have found that presence is associated with native species composition [91]. Studies of Horned Larks in other regions have found that this species nests in microsites well-protected from prevailing winds by individual bunchgrasses [92, 93], and nesting requirements could also explain the association between native cover and Horned Larks in our study. The most common native plant species in this study, the perennial bunchgrass purple needlegrass, forms a tussock different in structure from exotic annual grasses introduced to California. Plots with higher native cover tended to have higher plant species richness, and greater abundance of both annual and perennial forbs, both of which are typically shorter and more variable in stature than exotic annual grasses. Annual grasses provide a denser, more homogeneous canopy during the growing season, permitting less light and space between plants [94], and they tend to produce more aboveground biomass than perennials, which invest more heavily in root growth [95].

Other factors that could be responsible for higher occupancy where there is higher native cover include concealment from predators and food availability. The timing, diversity, and quality of food available for grassland birds likely differs in an exotic annual-dominated grassland habitat compared to the native perennial bunchgrass and forb dominated ecosystem that may have existed pre-European settlement [51]. Grassland specialist birds consume seeds, small insects, and other invertebrates, and recruit according to food availability and density [13, 54, 58].The vegetation structure and community influence the type, abundance, and density of insects, and therefore may mediate bird species composition and abundance [13, 96].

Vertical and horizontal vegetation structural properties are key factors used by birds to select breeding habitat, and grassland birds are thought to minimize interspecific competition by partitioning habitat based on utilization of different microsites with different structural properties [12]. Structural factors can affect the efficiency of nesting and foraging behavior [92, 97]. In our study, Horned Larks and Western Meadowlarks were positively associated with bare ground. This result is consistent with those of other studies that have found this species to be associated with less dense herbaceous vegetation, shorter grass, and more bare ground [12]. Contrary to expectations, Grasshopper Sparrow occupancy was not strongly associated with structural features of vegetation; these variables were less important than northerly aspect, flatter topography, and cover of native plants. Western Meadowlarks were also positively associated with greater cover of bare ground. In other studies, these species are found in areas with taller vegetation, more litter and less bare ground than Horned Larks [12, 98].

In our study, we also found that light- to-moderate livestock grazing is a compatible land use with grassland birds. All three species were at least tolerant of grazing by livestock. Horned Lark was found more often in plots where grazing was present. In fact, we found evidence that Horned Lark depend on conditions created by annual grazing: observations at Vasco Caves declined dramatically after the second year of the study, when livestock animals were removed for management reasons unrelated to our research. t. This indicates that changes associated with grazing removal can occur relatively rapidly after livestock are excluded.

All three species were associated with topographical features such as north-facing slopes, and the slope of the terrain. Both Western Meadowlarks and Grasshopper Sparrows preferentially used grasslands with more north-facing aspect, regardless of grazing status. Cooler, north-facing slopes in California grasslands tend to contain higher native cover than south-facing slopes [99, 100]; a pattern which was also evident in our dataset (Table 5). In previous studies from other regions, Grasshopper Sparrows were associated with nonnative vegetation [90], native plant species [56], and successional stage or structural characteristics [58, 91, 101–103]. Variability among geographic location of these studies is probably responsible for the differences among findings; grassland vegetation structure and species composition differs dramatically between regional types.

**Table 5:** Support for linear mixed models representing the effects grazing, northness, slope and native cover on vegetation structural characteristics in grasslands grazed by livestock in the northern Diablo Range of California. Models are ranked in decreasing order of support, and only models with Δ AIC_c_ < 4 are shown. Table includes the number of parameters (*K)* in each model, the change in AIC_c_ from the best performing model to all others and the AIC_c_ weight (*w*_*i*_*)* or strength of evidence for each model.

* *	Model	*K*	AIC_c_	Δ AIC_c_	*w*_*i*_
*Height*					
	Grazing + Native cover + Northness	7	611.09	0	0.72
	Grazing + Native cover + Northness + Slope	8	613.10	2.01	0.26
*Vertical heterogeneity*					
	Grazing + Native cover + Northness	7	179.85	0	0.17
	Grazing	5	179.92	0.08	0.16
	Grazing + Native cover + Northness + Slope	8	179.96	0.12	0.16
	Grazing + Native cover	6	180.35	0.50	0.13
	Grazing + Slope	6	180.71	0.87	0.11
	Grazing + Northness	6	180.78	0.94	0.10
	Grazing +Native cover + Slope	7	180.92	1.08	0.10
	Grazing + Northness + Slope	7	181.39	1.55	0.08
*Litter (%)*					
	Grazing + Slope	6	778.21	0	0.37
	Grazing + Northness + Slope	7	779.26	1.05	0.22
	Grazing + Native cover + Slope	7	779.54	1.33	0.19
	Grazing + Native cover + Northness + Slope	8	779.73	1.52	0.17
*Bare ground (%)*					
	Grazing + Native cover + Northness + Slope	8	750.27	0	0.80
*Native cover*					
	Grazing + Northness	6	945.48	0	0.57
* *	Grazing + Northness + Slope	7	946.08	0.60	0.43

Occupancy by all three species in this study was variable both between years and across sites. Neither landscape factors nor climate variables were included in this study, and probably account for some of the variation in occupancy. Factors such as grassland patch size [104], fragmentation [105, 106], habitat edges [107], and landscape structure [108], proximity to woodland areas [62], roads [109], or other human disturbance [110] can all have an influence on grassland birds via indirect effects such as predator abundance and brood parasitism [111], and climate can affect productivity and structural conditions [112]. Previous research in the same study area examined the effects of patch size and landscape-scale heterogeneity on these birds, and found that grassland songbird presence was correlated with larger patch sizes and low heterogeneity of land cover types [9]. Variability across years has also been documented in other studies [113]. While Horned Larks and Western Meadowlarks are not migratory in this region of Northern California, it is likely that they respond to year-to-year variation in vegetation and microsite conditions when selecting foraging and breeding sites.

Vegetation structure is well known to be a primary factor for grassland birds when they choose areas for nesting and foraging. An area with a patch of native bunchgrasses and forbs included in a California grassland dominated by nonnative annual grasses likely creates more variation in height and density of grasses in the native patch with interspersed bare ground.

In northeastern Oregon, Kennedy, DeBano et al. [46] compared bunchgrass prairie plant communities with a gradient of 47–99% native cover and found no difference in reproductive success metrics for grassland bird species (Western Meadowlark, Horned Lark), including nesting density and survival, clutch size and productivity. Another study in California that compared native and exotic dominated grasslands [56], found that Grasshopper Sparrows were associated with native bunchgrass cover.

Moderate intensity livestock grazing can create this preferable patchy grassland structure [10, 26], potentially even in highly invaded Mediterranean grassland that often has a dense layer of exotic annual grasses. We found Horned Larks prefer livestock-grazed grassland, and the association was less strong in areas with higher native plant cover.

A study in southeastern Arizona suggests insect prey abundance may be another reason grassland birds prefer areas with a mix of native and exotic plants [114]. Litt and Steidl [114] found increasing levels of exotic plant invasion have a strong negative effect on insect richness and overall abundance. Grassland bird species are known to primarily eat insects during the breeding season [115] which probably explains a higher presence in native grassland areas in California. However, when the Kennedy, DeBano et al. [46] study, located in fescue bunchgrass prairie in northeastern Oregon, compared different levels of nonnative plant cover there was no noticeable difference in insect abundance. Although this is a more arid bunchgrass area, the suggestion by Kennedy, DeBano et al. [46] that the greater impact of an increase in nonnative plants was the decrease in bare ground and the loss of easy foraging, indicated by a diet switch away from ground dwelling insects, might also hold true in our grassland system.

## Conclusion

Although habitat needs differ somewhat among the three bird species in this guild, our study indicates that flatter topography, higher native species abundance, and moderate levels of livestock grazing allow for or support all three species in Mediterranean annual-dominated grasslands in California. However, much of the flat, native-dominated Mediterranean grassland habitat type has already been converted to agricultural or urban land uses in California, and that which remains continues to be at very high risk of degradation or conversion [5]. Furthermore, Rao et al. [9] found that larger grassland patch size is important for these bird species. Taken together, there is strong evidence that protection of large blocks of remaining habitat may be an urgent need for grassland bird conservation. With respect to grassland management, livestock grazing reduces litter and creates bare patches through removal of biomass each growing season, which is beneficial for native plant species and encourages vegetation structure preferred by all three birds. Grazing is therefore a compatible or supportive management activity. But given the extent of conversion, the degree to which many grasslands have been invaded by exotic annual grasses, and the low abundances found across the study area especially in highly invaded sites, active habitat restoration (e.g., seeding, planting, management of invasive or undesirable species, targeted grazing, fire) in grasslands to enhance native plant cover and vegetation structure may also be needed in addition to grazing to support this unique and rapidly declining bird guild over the long term.

## Supporting information

S1 File Vegetation, land cover, topography, grassland bird presence, and grazing data are shown for each year, 2004–2011; model variables, bird species codes, and bird species abundance are also provided. (XLSX)
